# A Metagenomic Analysis of Mosquito Virome Collected From Different Animal Farms at Yunnan–Myanmar Border of China

**DOI:** 10.3389/fmicb.2020.591478

**Published:** 2021-02-08

**Authors:** Muddassar Hameed, Abdul Wahaab, Tongling Shan, Xin Wang, Sawar Khan, Di Di, Liu Xiqian, Jun-Jie Zhang, Muhammad Naveed Anwar, Mohsin Nawaz, Beibei Li, Ke Liu, Donghua Shao, Yafeng Qiu, Jianchao Wei, Zhiyong Ma

**Affiliations:** Shanghai Veterinary Research Institute, Chinese Academy of Agricultural Sciences, Shanghai, China

**Keywords:** mosquito, mosquito virome, metagenomics, viral community, animal farm

## Abstract

Metagenomic analysis of mosquito-borne and mosquito-specific viruses is useful to understand the viral diversity and for the surveillance of pathogens of medical and veterinary importance. Yunnan province is located at the southwest of China and has rich abundance of mosquitoes. Arbovirus surveillance is not conducted regularly in this province particularly at animal farms, which have public health as well as veterinary importance. Here, we have analyzed 10 pools of mosquitoes belonging to *Culex tritaeniorhyncus*, *Aedes aegypti*, *Anopheles sinensis*, and *Armigeres subalbatus* species, collected from different animal farms located at Yunnan province of China by using metagenomic next-generation sequencing technique. The generated viral metagenomic data reveal that the viral community matched by the reads was highly diverse and varied in abundance among animal farms, which contained more than 19 viral taxonomic families, specific to vertebrates, invertebrates, fungi, plants, protozoa, and bacteria. Additionally, a large number of viral reads were related to viruses that are non-classified. The viral reads related to animal viruses included parvoviruses, anelloviruses, circoviruses, flaviviruses, rhabdoviruses, and seadornaviruses, which might be taken by mosquitoes from viremic animal hosts during blood feeding. Notably, the presence of viral reads matched with Japanese encephalitis virus, Getah virus, and porcine parvoviruses in mosquitoes collected from different geographic sites suggested a potential circulation of these viruses in their vertebrate hosts. Overall, this study provides a comprehensive knowledge of diverse viral populations present at animal farms of Yunnan province of China, which might be a potential source of diseases for humans and domestic animals.

## Introduction

Mosquitoes are the most frequently observed arthropod vectors, with the potential to transmit several viruses that cause diseases of significant human health impact, such as dengue fever, yellow fever, Zika fever, West Nile fever, and Japanese encephalitis (JE) (Dash et al., 2013; Napp et al., 2018; Sukhralia et al., 2019). These geographically endemic diseases are responsible for enormous economic burdens and global health concern in both developing and developed countries (LaBeaud et al., 2011; Gould et al., 2017; Mayer et al., 2017; Ferguson, 2018). In addition to these viruses associated with vertebrate infections, several viruses belonging to diverse viral families have been identified in mosquito populations (Li et al., 2015; Xia et al., 2018). Although these viruses do not have direct effect on human and animal health, they can modulate the transmission of viruses that are pathogenic (Vasilakis and Tesh, 2015; Hall et al., 2016). Recently, metagenomic next-generation sequencing (mNGS) analysis provides a better way to understand the diversity and abundance of mosquito-borne pathogens and novel agents to assess the epidemiology of emerging and reemerging mosquito-borne pathogens (Shi et al., 2017; Nanfack Minkeu and Vernick, 2018).

Mosquitoes are widely present at animal farms, and their blood-feeding behavior (feeding on myriad hosts) makes them an efficient vector for many pathogens of human and animals (Molaei et al., 2006; Colpitts et al., 2012). Therefore, mosquitoes that can be easily captured could serve as a sentinel model to efficiently survey virus burden in animal farms instead of sampling blood from individual animal that is laborious and time-consuming. Furthermore, as reported in previous studies, viruses have an extraordinary evolutionary potential leading to the emergence of new pathogen strains that can cause severe diseases in human and animals (Drake and Holland, 1999; Sanjuán et al., 2010; Greenwood et al., 2018). Because of these reasons, the diversity and richness of mosquito viral flora should be analyzed for the surveillance of viral population burden at farm level that have a potential to spread to humans and domestic animals.

Yunnan province is located at the southwest of China, adjacent to Myanmar, Laos, and Vietnam. Ecologically, Yunnan has tropical and subtropical climates and provides favorable conditions for the proliferation and dissemination of mosquitoes and conducive to the maintenance and transmission of existing arboviruses, as well as the emergence of new arboviruses. These factors highlight the need for the comprehensive surveillance of virus diversity in mosquitoes. In the last few years, multiple studies have been conducted in different regions of China and report several unclassified and novel mosquito-associated viral sequences, such as Wuhan mosquito virus, Xinzhou mosquito virus, Zhejiang mosquito virus, Zhee mosquito virus, Wutai mosquito virus, *Culex* (*Cx.*) *tritaeniorhyncus* rhabdovirus, etc. (Li et al., 2015; Shi et al., 2015, 2016; Atoni et al., 2018; Xia et al., 2018). Recently, Xiao et al. (2018a; 2018b) performed a metagenomic analysis of mosquitoes from Yunnan province and reported the presence of a variety of insect and human viruses including dengue virus (DENV), Zika virus (ZIKV), and JE virus (JEV). However, information regarding the mosquitoes present at different animal farms of this province is lacking. Therefore, in this study, we collected mosquitoes from animal farms in the border areas of Yunnan province for surveillance of virome burden harbored by animal farm mosquitoes.

For a better understanding of mosquito virome of animal farms, we have utilized the mNGS, cell culture, and polymerase chain reaction (PCR)–based approach to investigate the abundance and diversity of viruses in 10 pools of mosquitoes, belonging to *Cx. tritaeniorhyncus*, *Aedes (Ae.) aegypti*, *Anopheles (An.) sinensis*, and *Armigeres (Ar.) subalbatus* species, collected from different animal farms located at Yunnan–Myanmar border of China as a part of arboviruses surveillance program. We found the abundance of viral reads matched with a broad range of viruses that can infect vertebrates and invertebrates. Overall, these results revealed diverse virome burden in mosquitoes present at animal farms.

## Materials and Methods

### Mosquito Collection

**FIGURE 1:**
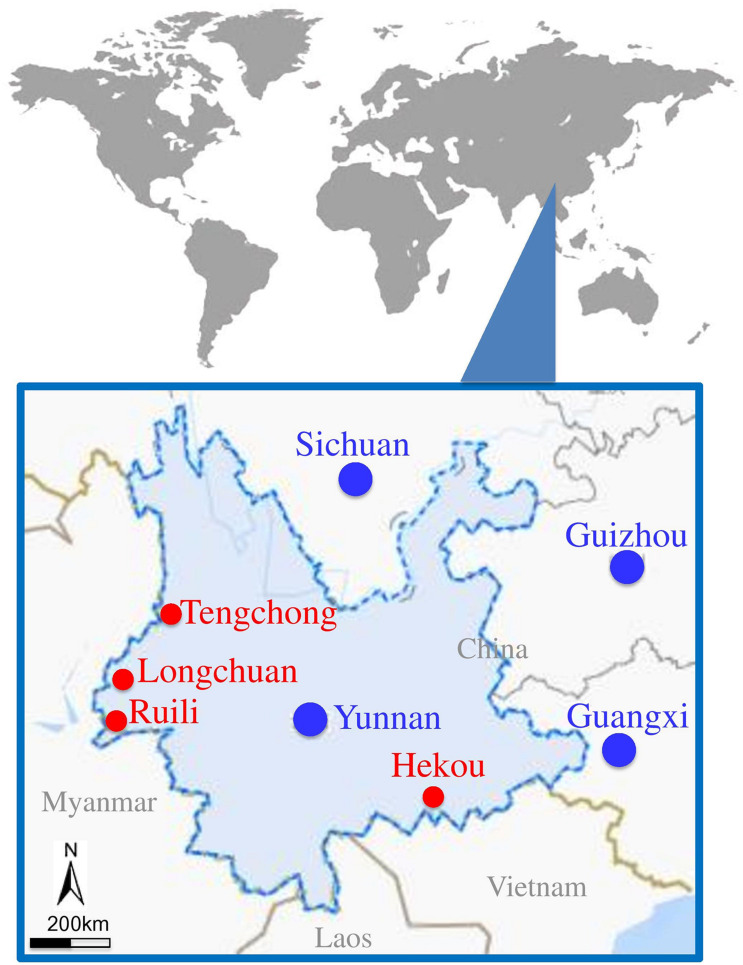
Locations of mosquito collection sites located at border area of Yunnan–Myanmar. Mosquito collected areas are presented with red circles.

Mosquitoes were collected from different animal farms located at Yunnan–Myanmar border of China (Figure 1 and Supplementary Table 1) using black ultraviolet (UV)–light traps (12 V, 300 mA; Photocatalytic Technology Mosquito Catcher Device, Electrical Technology, Guangdong, China) during June and July 2018. Five black UV-light traps were set for each farm: three placed in different barns and two under the eaves outside. All traps were suspended 2 m above the ground and operated one night from 9:00 PM to 6:00 AM next morning. Among the female mosquitoes collected, approximately 10–25% of mosquitoes were engorged with blood, and we tried to use most of the engorged female mosquitoes for metagenomic analysis. The collected mosquitoes were morphologically identified at species level (*Handbook for Classification and Identification of Main Vectors*) and subsequently confirmed by DNA barcoding using the cytochrome oxidase subunit I (COI) gene from the mitochondrial genome, as described previously (Folmer et al., 1994). The species including *Cx. tritaeniorhyncus*, *An. sinensis*, *Ar. subalbatus*, and *Ae. aegypti* were grouped into 10 sample pools (B3, C1, C2, C3, D2, E1, F1, G1, G2, and G3) for metagenomic analysis (Table 1 and Supplementary Table 2). Each grouped sample containing approximately 100 adult female mosquitoes was grinded manually using a glass homogenizer with 2 mL of Dulbecco modified Eagle medium (Thermo Fisher Scientific, Carlsbad, CA, United States) containing 100 U/mL penicillin and 50 U/mL streptomycin. The homogenized samples were centrifuged at 13,000×*g* for 30 min at 4°C to remove mosquito debris, and supernatant was collected and then stored at −80°C until further use. Half of the supernatant was used to extract viral nucleic acid for metagenomic analysis, and the remaining half was used for viral isolation.

**TABLE 1:** Mosquito samples used for metagenomic analysis and generated data.

Sample name	Locations	Farm	Species	Numbers	Total reads	Average read length	Viral reads
B3	Ruili-2	YPY	*Cx. tritaeniorhyncus*	100	12,010,062	124	2,568,397
C1	Ruili-2	YPY	*Ae. aegypti*	100	32,946,938	118	7,530,960
C2	Tengchong	BFL	*Cx. tritaeniorhyncus*	100	16,681,916	124	2,823,450
C3	Tengchong	BFL	*Ae. aegypti*	100	6,546,774	128	33,962
D2	Tengchong	BFL	*An. sinensis*	99	27,061,464	121	700,311
E1	Longchuan	YEY	*Cx. tritaeniorhyncus*	100	4,915,422	126	620,559
F1	Hekou	MYJ	*Cx. tritaeniorhyncus*	100	12,615,384	123	93,942
G1	Hekou	MYJ	*Ae. aegypti*	107	41,329,364	122	4,548,211
G2	Hekou	MYJ	*Ar. subalbatus*	96	2,740,686	128	9,655
G3	Hekou	MYJ	*An. sinensis*	107	15,600,482	125	618,527
Total/Average				1,009	172,448,492	122	19,547,974

### Nucleic Acid Extraction and Reverse Transcription

To remove contaminating host genomic DNA and free nucleic acid, 14 U Turbo DNase (Ambion, Austin, TX, United States), 25 U Benzonase Nuclease (Novagen, San Diego, CA, United States), 20 U RNase-I (Fermentas, Ontario, Canada), and 10 × DNase buffer (Ambion) were added to 127 μL of supernatant to make a final volume of 150 μL, followed by digestion at 37°C for 1 h. Total viral nucleic acid in the obtained products was isolated using Nucleic Acid Extraction kit (Hangzhou Bioer Technology, Hangzhou, China) according to the manufacturer’s instructions. Viral RNA was reverse-transcribed using anchored random primers and Superscript III reverse transcriptase (Invitrogen, Carlsbad, CA, United States). The random primers were added separately to the viral nucleic acid and incubated at 65°C for 5 min and then transferred to ice for 5 min for denaturation (Hameed et al., 2020). To get the final reverse-transcribed product, 40 U of RNase OUT (Invitrogen), 200 U of SuperScript III reverse transcriptase (Invitrogen), 1 μL of 0.1 M dithiothreitol (Invitrogen), 1 μL of 10 mM dNTPs (TaKaRa, Dalian, China), 4 μL of 5 × first-strand buffer (Invitrogen), and RNase-free H_2_O (TaKaRa) were added to a final volume of 20 μL and incubated at 25°C for 10 min, followed by 50°C for 60 min and then 75°C for 10 min.

### Synthesis of Double-Strand cDNA

RNase H (TaKaRa) was added to the obtained reverse-transcribed products to degrade free RNA. To synthesize double-strand cDNA (dscDNA), anchored random primers were added and incubated at 65°C for 5 min and then placed on ice for 5 min for denaturation (Hameed et al., 2020). After this, 1 μL of Klenow fragment (TaKaRa), 1 μL of 10 mM dNTPs (TaKaRa), 2 μL of 10 × Klenow buffer (TaKaRa), and 6 μL of ddH_2_O (TaKaRa) were added and incubated at 37°C for 60 min, followed by an incubation at 75°C for 10 min. To remove phosphates and the free single-strand nucleic acid in the dscDNA reaction, 0.5 μL of Exonuclease I (TaKaRa), 1 μL of alkaline phosphatase (TaKaRa), 5 μL of 10 × phosphatase buffer (TaKaRa), and 24 μL of DEPC H_2_O (TaKaRa) were added and incubated at 37°C for 60 min, followed by an incubation at 75°C for 10 min.

### Sequence-Independent, Single-Primer Amplification, and Purification of PCR Products

The dscDNA was amplified using sequence-independent, single-primer amplification. The 50 μL reaction mixture comprised 10 μL of dscDNA, 2 μL of barcode primer, 1 μL of AccuPrime Taq DNA polymerase (Invitrogen), and 37 μL of ddH_2_O (TaKaRa) (Hameed et al., 2020). The PCR conditions were as follows: 95°C for 20 s, 54°C for 20 s, 68°C for 70 s, and a final extension at 68°C for 7 min. PCR products were purified using a PCR purification kit (QIAGEN, Hilden, Germany) and eluted in 30 μL of TE buffer (100 mM Tris–HCl, 10 mM EDTA, pH 8.0) (Promega, Madison, United States).

### Metaviral Sequencing

The purified PCR products from 10 samples were sent to the BGI Genomics (BGI, Shenzhen, China) for Illumina sequencing. To obtain ∼180 bp DNA fragments, PCR products were ultrasonicated, and then dATPs and Klenow fragments were added to produce 3 dA overhangs. To establish genomic DNA libraries, DNA fragments were bound to Illumina adaptors and amplified using PCR with adaptor primers. Amplicons were ligated to flow cells to which fluorescently labeled dNTPs were added. DNA sequences were identified using the sequencing-by-synthesis method (SBS, Illumina). Base calling was performed by the program GAPipeline (BGI), with default settings. No-calling reads and adaptor sequences were removed. The remaining sequences were assembled into contiguous sequence (contig) using SOAP *de novo* software (BGI). Contigs and sequences longer than 100 bp were defined as significant data for further *in silico* analysis.

### Computational Analysis

Contigs and sequences were aligned using Blastx and Blastn with the non-redundant and viral reference sequences in the GenBank database^1^. Blast hits with an *E* value of ≤10e–5 were considered significant. After removing the bacterial and eukaryotic sequences, the virus-like sequences were analyzed.

### Passage of Mosquito Supernatants on C6/36 and BHK-21 Cells

The mosquito *Ae. albopictus* C6/36 cell line and the baby hamster kidney cell line (BHK-21) were used for passage of mosquito supernatants to detect the presence of viruses, as described previously (Xiao et al., 2018a, b). Briefly, the supernatants of homogenized mosquito pools as mentioned above were filtered through 0.22 μm nitrocellulose filter paper and inoculated onto the cells followed by incubation for 2 h to allow virus adsorption. After addition of fresh medium, the cells were incubated at 28°C for C6/36 cells and at 37°C for BHK-21 cells and monitored daily for the development of cytopathic effect (CPE) until 7 days postinfection. The inoculated cells were blindly passaged for three to six times until CPE appearance. The supernatants harvested from the CPE-positive C6/36 cells were further inoculated onto BHK-21 cells for detection of the presence of infectious viruses. All experiments for the virus isolation were performed in biosafety level 2 cell culture laboratory established at Shanghai Veterinary Research Institute, China.

### Detection of Arboviruses in CPE-Positive Cells

BHK-21 and C6/36 cells were inoculated with the supernatant harvested from the CPE-positive cells and incubated for 36 h. The presence of arboviruses in the inoculated BHK-21 and C6/36 cells was detected by quantitative real-time reverse transcription–PCR (qRT-PCR), immunofluorescence assay (IFA), and Western blot. For qRT-PCR assay, primers specific to amplify the target genes of 12 arboviruses including JEV, Getah virus (GETV), Bunyavirus, Banna virus, *Cx.* flavivirus (CxFV), Chickengunya virus, Kadipiro virus, Liaoning virus, Yunnan orbivirus, DENV, ZIKV, and West Nile virus (WNV) were designed based on the respective sequences deposited in GenBank and synthesized (Supplementary Table 3). Viral nucleic acids were extracted from the CPE-positive cells using a virus nucleic acid extraction kit (Bioer Technology) and immediately reverse transcribed by using PrimeScript^TM^ RT Master Mix (Takara, Tokyo, Japan). PCR amplification was carried out using this cDNA with Q5 DNA high-fidelity polymerase (New England BioLabs) following the manufacturer instructions. The PCR products were gel purified using QIAquick agarose gel DNA extraction kit (Hilden, Germany), as per the kit’s protocol, and commercially sequenced at Shanghai Sunny Biotechnology Co., Ltd., China. For detection of the presence of JEV and GETV in the inoculated BHK-21 cells, IFA and Western blot analysis were performed using antibodies specific to JEV NS3 protein (Deng et al., 2011) and GETV E2 protein (F3699A, Feimobio, China), respectively, as previously described (Deng et al., 2011; Zhu et al., 2013).

### Phylogenetic Analysis

Based on the alignment data of viral contigs and the match positions of contigs with JEV, PPV2, PPV3, Hubei mosquito virus 2 (HMV2), and GETV, the previously available genomes of relevant viruses were retrieved from GenBank. These downloaded sequences were trimmed according to the length of JEV, GETV, PPV2, PPV3, and HMV2 sequences generated in the present studies. Multiple sequence alignment of these sequences was performed using CLUSTAL W, as implemented in BioEdit software (Hall, 1999), and inspected manually. The complete NS2B and partial NS5 gene sequences of JEV, partial E2 gene of GETV, NS1 gene of PPVs, and hypothetical protein 1 gene of HMV2 were used for Bayesian phylogeny reconstructions that were performed in a Bayesian framework with BEAST 2 (Bouckaert et al., 2014). The Bayesian phylogenetic trees were extracted by TreeAnnotator and then visualized and finished in FigTree^2^.

## Results and Discussion

### Metagenomic Analysis of Mosquito Viromes Captured From Different Animal Farms

To survey the virus burden in mosquitoes present at various animal farms, a total of 4,576 live or freshly dead mosquitoes (male + female) were collected from four animal farms at geographically diverse locations of Yunnan province (Figure 1 and Supplementary Table 1). The mosquito species were morphologically identified at species level (Zhou and Chu, 2019) and subsequently confirmed by DNA barcoding using COI gene (Supplementary Figure 1). The identified mosquitoes were classified into seven species: *Cx. tritaeniorhyncus* (2,607), *Cx. quinquefasciatu* (8), *An. sinensis* (343), *An. minimus* (6), *Ar. subalbatus* (101), *Ar. obturbans* (10), and *Ae. aegypti* (1,501), with variable abundance among different farms (Supplementary Table 2). The *Cx. tritaeniorhyncus* was the most abundant species accounting for 56.9%, followed by *Ae. aegypti* (32.8%), *An. sinensis* (7.5%), and *Ar. subalbatus* (2.2%). These four species with relatively high abundance were divided into 10 groups according to the different species from different animal farm (Table 1) and subjected to mNGS. After mNGS, a total of 172,448,492 reads with 122 bp average read length were generated from different mosquito samples, of which 19,547,974 reads were identified as viral reads (Table 1).

**FIGURE 2:**
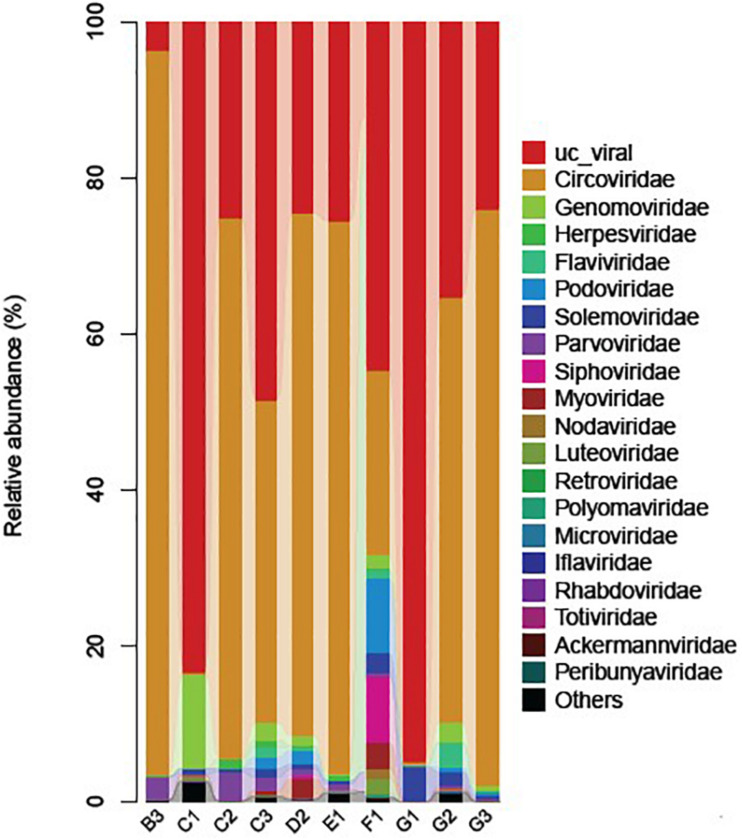
Stacked bar graph of virus families (color coded) identified in the viromes of the mosquitoes collected from different animal farms. “UC_viral” shows the presence of viral reads that were classified as non-classified virus sequences.

Subsequent blast analysis of the viral reads revealed that the mosquitoes collected from animal farms harbored the viral sequences mainly related to 19 viral taxonomic families with variable prevalence, including *Circoviridae*, *Genomoviridae*, *Herpesviridae*, *Flaviviridae*, *Podoviridae*, *Solemoviridae*, *Parvoviridae*, *Siphoviridae*, *Myoviridae*, *Nodaviridae*, *Luteoviridae*, *Retroviridae*, *Polydnaviridae*, *Microviridae*, *Iflaviridae*, *Rhabdoviridae*, *Totiviridae*, *Ackermannviridae*, and *Peribunyaviridae* (Figure 2). These data suggested a high diversity of viral communities carried by animal farm mosquitoes, which may have a potential to infect a wide range of hosts including vertebrate, invertebrate, plant, bacteria, algae, and fungi (Supplementary Figure 2 and Supplementary Table 4).

Noticeably, a large number of reads related to the non-classified viruses were observed among all samples (Figure 2), presumably belonging to the unexplored viruses. Identification and characterization of these non-classified viruses present in mosquitoes may yield important insights into the evolutionary history of other significant viruses. As mentioned in previous studies that viruses have extraordinary mutation potential, which can lead to the emergence of new vertebrate diseases (Sanjuán et al., 2010; Greenwood et al., 2018; Kretova et al., 2018), the discovery and surveillance of these strains thus could be helpful to prevent future outbreaks.

### Family *Flaviviridae*, Japanese Encephalitis Virus

**FIGURE 3:**
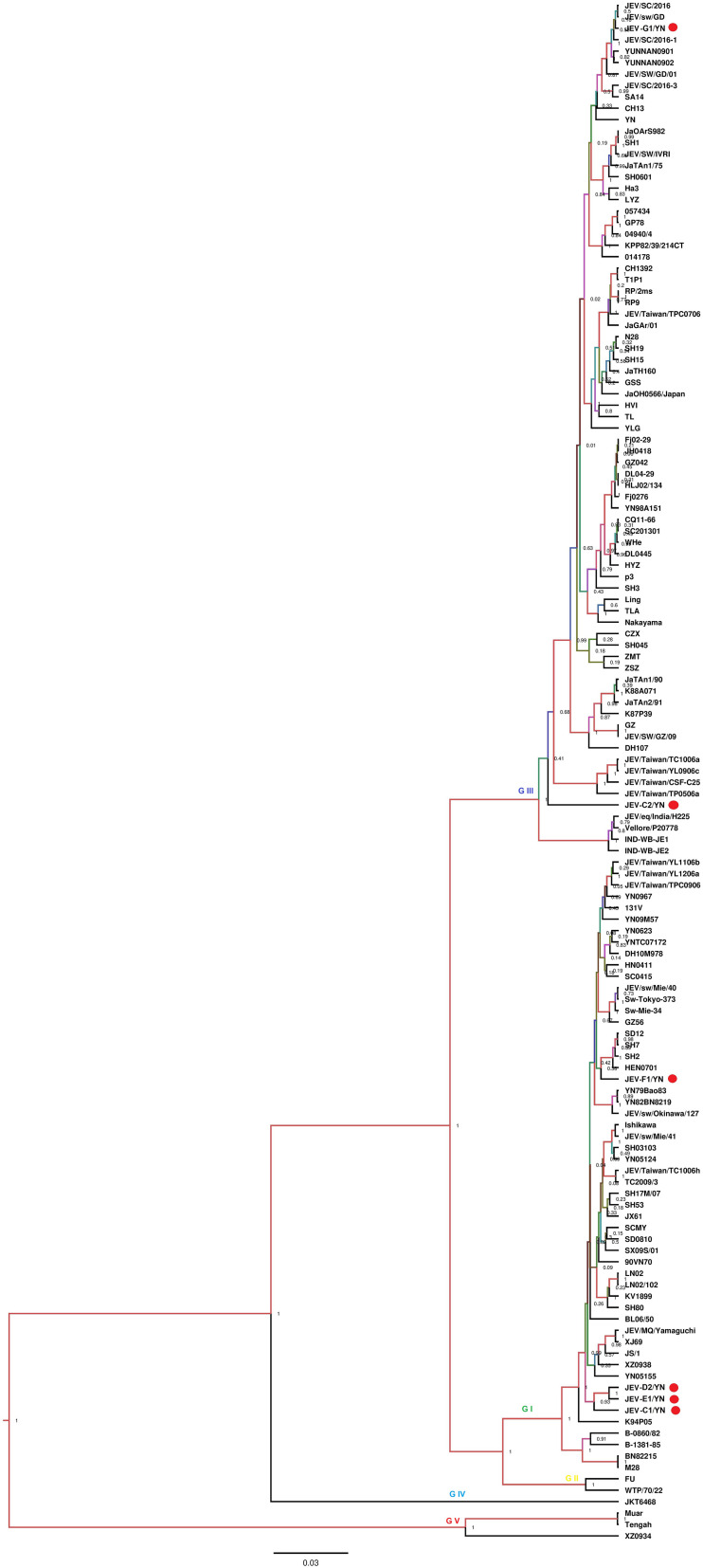
Phylogenetic analysis of nucleotide sequences of partial NS5 gene of JEV detected in the mosquito samples. Different JEV sequences identified in the present study are labeled with red circle.

**FIGURE 4:**
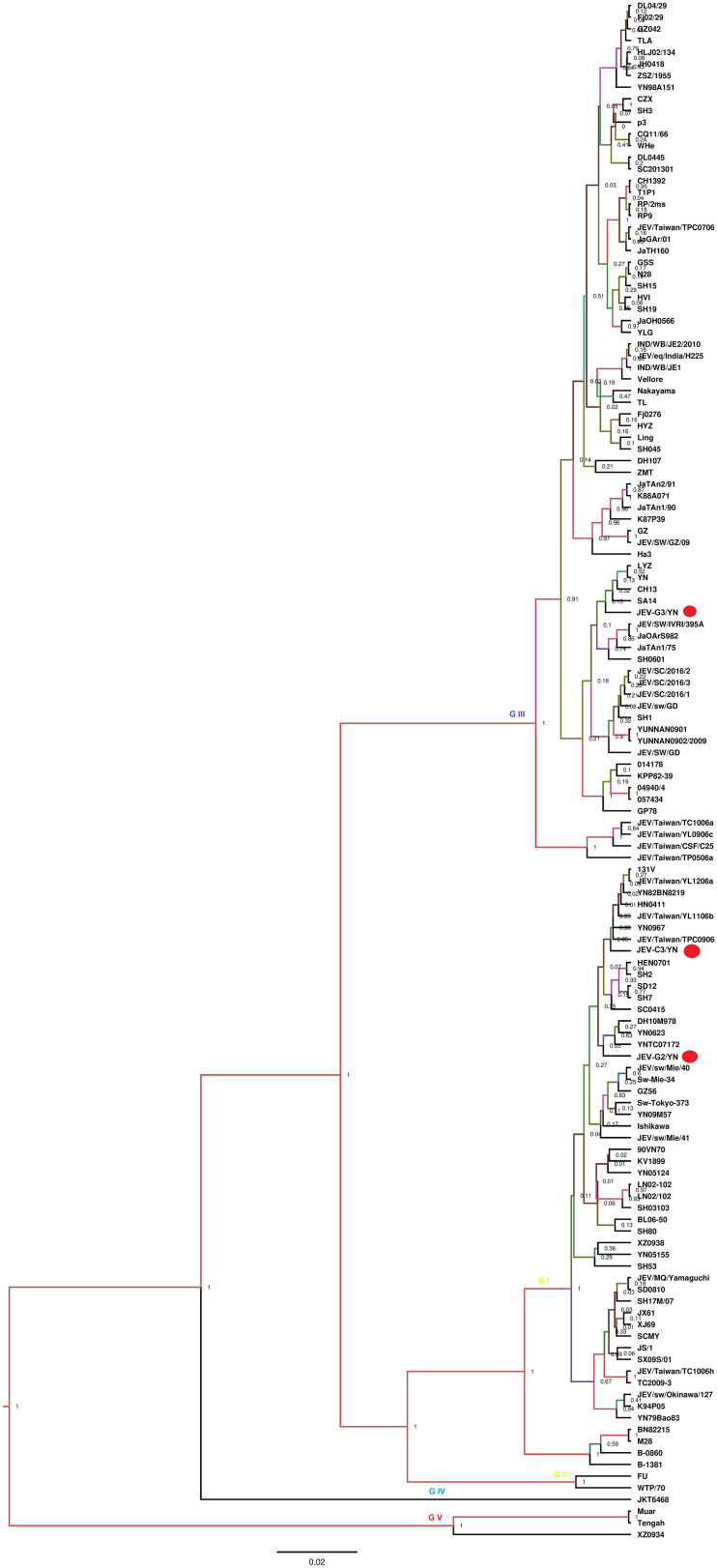
Phylogenetic analysis of nucleotide sequences of complete NS2B gene of JEV detected in the mosquito samples. Different JEV sequences identified in the present study are labeled with red circle.

Nine of 10 samples (C1, C2, C3, D2, E1, F1, G1, G2, and G3) contained reads related to JEV that harbors a genome consisting of three structural and seven non-structural protein genes (C-prM-E-NS1-NS2A-NS2B-NS3-NS4A-NS4B-NS5). Blast results showed that these sequences were most similar to JEV strains that are identified previously in China. These reads were assembled into variable number of contigs (1–3) for different samples with 624–8,068 bp in length, which shared 94.4–99.4% nucleotide identity to the referenced strains (Table 2). The JEV sequence detected in each mosquito sample was named accordingly (Table 2). A partial NS5 gene from the detected JEV-C1/YN, JEV-C2/YN, JEV-D2/YN, JEV-E1/YN, JEV-F1/YN, and JEV-G1/YN sequences and a complete NS2B gene from the detected JEV-C3/YN, JEV-G2/YN, and JEV-G3/YN sequences were further aligned with 129 publicly available JEV genomes (Supplementary Table 5) to construct Bayesian phylogenetic tree (Figures 3, 4). The Bayesian phylogenetic analysis showed that the JEV sequences detected in the present study were closely related to the isolates from Yunnan province as well as other JEV strains previously reported from China (Figures 3, 4). Yunnan province is located in the southwest of China and is still epidemic area for JEV. JEV genotype I (GI) and genotype III (GIII) have been reported from Yunnan province, and GI is the most common isolated genotype (Zheng et al., 2012; Xiao et al., 2018a). In our study, JEV sequences detected in six samples (C1, C3, D2, E1, F1, and G2) were clustering with JEV GI isolates reported from China, whereas the sequences from three samples (C2, G1, and G3) were most closely matched with JEV GIII strains (Figures 3, 4).

**TABLE 2:** Information of assembled contigs of JEV.

Samples	Contigs	No. of reads	Matched strains (Genbank no.)	Query cover	Identity (%)	*E*-value	Covered range	Covered viral gene	Sequences for phylogenetic analysis	Name of strain
C1	C1-1	93	JF706281	100	99.39	0	4,348–5,171	NS2B-NS3		JEV-C1/YN
	C1-2	107	JF706267	100	97.60	0	9,217–10,301	NS5	NS5	
C2	C2-1	83	KU904395	99	94.39	0	4,291–5,074	NS2B-NS3		JEV-C2/YN
	C2-2	150	MH385014	100	95.93	0	7,311–8,486	NS4B-NS5		
	C2-3	105	MH385014	100	95.66	0	9,112–10,239	NS5	NS5	
C3	1	473	MH385014	100	98.18	0	4,347–5,446	NS2B-NS3	NS2B	JEV-C3/YN
D2	1	3,910	HM228921	99	98.36	0	2,271–10,337	E-NS1-NS2A-NS2B-NS3-NS4B-NS5	NS5	JEV-D2/YN
E1	E1-1	124	JF706282	100	98.84	0	4,339–5,112	NS2B-NS3		JEV-E1/YN
	E1-2	170	MH385014	100	96.62	0	9,541–10,488	NS5-3′UTR	NS5	
F1	1	520	JF706268	100	95.86	0	9,400–10,268	NS5	NS5	JEV-F1/YN
G1	G1-1	206	MH753128	99	99.02	0	1,743–3,773	E-NS1-NS2A		JEV-G1/YN
	G1-2	215	KU323483	100	98.68	0	4,181–6,071	NS2A-NS2B-NS3		
	G1-3	501	MN639770	100	98.96	0	7,283–10,348	NS4B-NS5	NS5	
G2	G2-1	207	JF706267	100	97.76	0	4,369–4,992	NS2B-NS3	NS2B	JEV-G2/YN
	G2-2	269	FJ495189	100	95.65	0	8,423–9,497	NS5		
G3	1	429	JN381861	100	96.04	0	4,180–5,793	NS2A-NS2B-NS3	NS2B	JEV-G3/YN

**FIGURE 5:**
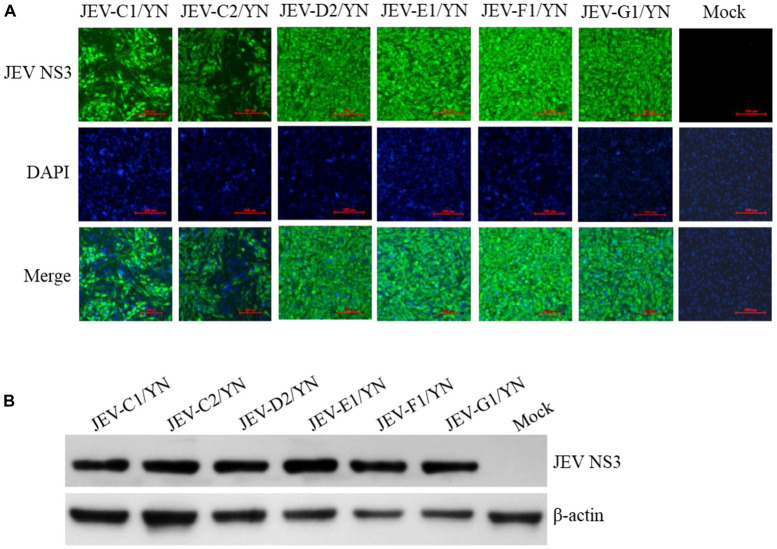
Detection of JEV in BHK-21 cells. BHK-21 cells were inoculated with the supernatants harvested from the CPE-positive BHK-21 cells inoculated with mosquito samples and incubated for 36 h. The presence of JEV was detected by IFA **(A)** and Western blot **(B)** with antibodies specific to JEV NS3 protein. Nuclei were stained with DAPI.

The samples (C1, C2, C3, D2, E1, F1, G1, G2, and G3) that were positive for JEV reads were further inoculated onto BHK-21 cells to detect the presence of infectious JEV. After four to five blind passages, CPE appeared in the cells inoculated with C1, C2, D2, E1, F1, and G1 samples, but not with C3, G2, and G3 samples. To detect the presence of JEV in the CPE-positive cells, BHK-21 cells were inoculated with the supernatants harvested from the CPE-positive cells and subjected to IFA, Western blot, and qRT-PCR analysis. JEV was detectable in the inoculated cells, as demonstrated by IFA (Figure 5A) and Western blot (Figure 5B) with antibodies specific to JEV NS3 protein. Furthermore, the presence of JEV in the inoculated BHK-21 cells was confirmed by qRT-PCR with primers specific to JEV NS5 gene (Supplementary Table 6). These data suggested the presence of infectious JEV in C1, C2, D2, E1, F1, and G1 samples. Although the mNGS data showed that C3, G2, and G3 samples were positive for JEV, no CEP was produced in the cells inoculated with these samples. These results were similar to the previous observation that JEV detected by PCR from mosquito samples may not be isolated after culturing on cells (Wang et al., 2020). A possible explanation is that the metagenomic analysis was able to detect the presence of JEV genes even if infectious virus was not present.

JEV causes acute viral encephalitis in humans (Campbell et al., 2011) and reproductive disorders in pigs (Ricklin et al., 2016). The JEV enzootic transmission cycle is maintained in nature by invertebrate (mosquitoes) and vertebrate (pigs/birds) hosts (Wang and Liang, 2015). Three genotypes of JEV have been isolated in China, including GI, GIII, and genotype V (Liang et al., 2018). JEV GIII was the most commonly circulating strain in China before 2000, whereas recently it has been noticed that GI is displacing GIII and has been isolated from cerebrospinal fluid of patients and mosquitoes in different provinces of China including Yunnan province (Liang et al., 2018; Xiao et al., 2018b).

JE is still endemic in China, and JE cases occur during every month; however, the case reports begin to increase in May, peak in July and August, and decrease in September (Gao et al., 2010; Pan et al., 2016; Zhang et al., 2019). In the past, it had been experienced that a small number of JE cases were reported in the west and north of China, whereas a large number of cases every year occurred in the east and southwest of China (Zheng et al., 2012). Yunnan province is located in the southwest of China, where it is still epidemic area for JEV (Feng et al., 2016). In the present study, of nine samples harboring JEV sequences, six samples contained JEV sequences mostly matched with GI strains, in agreement with a previous study conducted in Yunnan province, in which GI is identified as the dominant genotype (Xiao et al., 2018a). The previous studies indicate that GI strains have higher replication efficiency in birds and pigs than GIII strains (Fan et al., 2019; Li et al., 2020), and the emergence and continuous circulation of GI strains could be permanent threats for pigs as well as for poultry. Development of precautionary measures, such as GI vaccine, in advance is needed to avoid future outbreaks due to this newly emerging genotype (Wei et al., 2019).

In the present study, the mosquito samples were collected from animal farms, which also comprised pig herds (Supplementary Table 1). The data generated in this study suggested that JEV GI was also more prevalent than GIII in the animal farms of Yunnan province. As an important pathogen for swine, JEV infection causes abortion and stillbirth in sows, death of piglets, and aspermia in boars, which leads to severe economic losses to pig industry (Yuan et al., 2016; Mansfield et al., 2017). An inactivated (P3) and live attenuated (SA-14-14-2) JEV vaccines were developed in China since 1968 and 1988, respectively (Gao et al., 2010; Yu, 2010). At present, both vaccines are used all over China, and the vaccine program is especially credited of the reduction in JE cases that have been observed recently. The presence of both JEV GI and GIII in mosquitoes indicates that JEV is continuously circulating in Yunnan province especially in farm animals from where mosquitoes take up during blood feeding, which is a potential threat for humans, as well as for pig industry. Therefore, an enhanced surveillance system is needed along with precautionary measures such as regular vaccination and mosquito control to avoid future outbreaks.

### Family *Togaviridae*, Getah Virus

**FIGURE 6:**
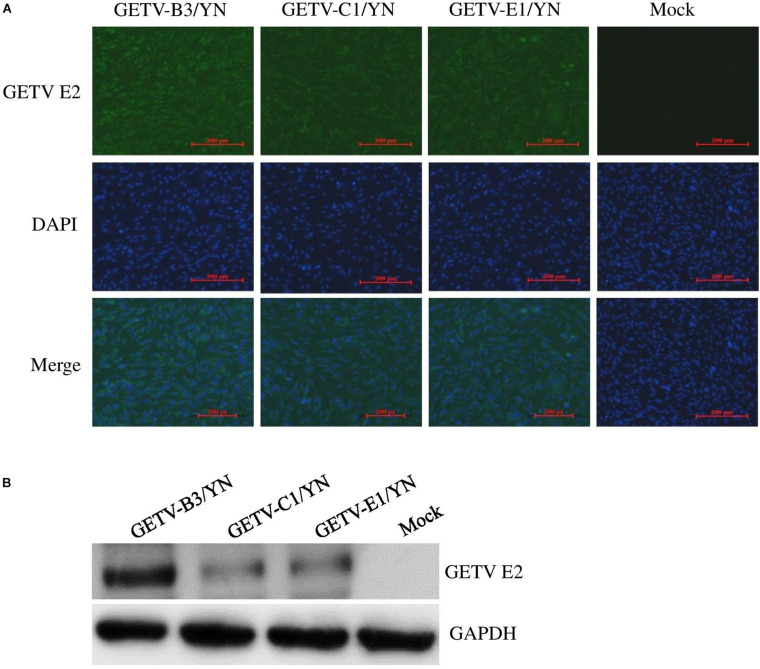
Detection of GETV in BHK-21 cells. BHK-21 cells were inoculated with the supernatants harvested from the CPE-positive C6/36 cells inoculated with mosquito samples and incubated for 36 h. The presence of GETV was detected by IFA **(A)** and Western blot **(B)** with antibodies specific to GETV E2 protein. Nuclei were stained with DAPI.

**FIGURE 7:**
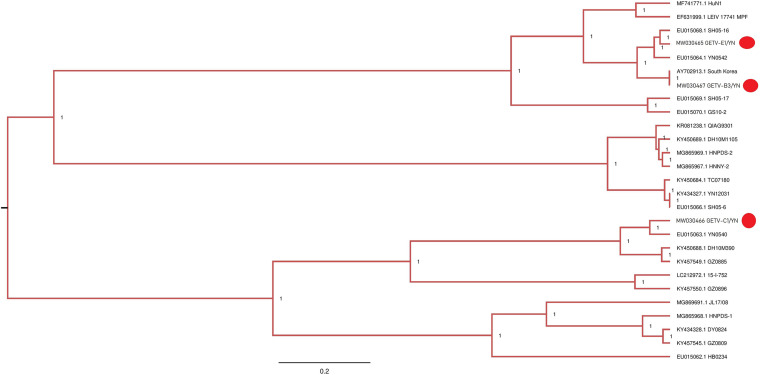
Phylogenetic analysis of partial nucleotide sequences of E2 gene of GETV detected in mosquito samples. Different GETV sequences identified in the present study are labeled with red circle.

During this arbovirus surveillance from animal farms, the virus burden in the collected mosquitoes was detected by using mNGS in combination with cell culture methods. Although metagenomic analysis allows the simultaneous identification of viruses from a single mosquito in a single reaction (Hall-Mendelin et al., 2013), this method has disadvantages compared to other molecular methods of virus detection. For example, it is less sensitive than qRT-PCR for the detection of samples with low virus titers, particularly when using mosquitoes as samples. Therefore, we employed the cell culture and qRT-PCR approaches in addition of mNGS to retrieve the complete mosquito virome, including viruses that exist at very low copy numbers, whereupon the results can be interpreted. C6/36 cells that are highly permissive to numerous arboviruses (White, 1987) were inoculated with the supernatant of each mosquito sample and passaged blindly until CPE appearance. Among 10 samples inoculated, eight samples (B3, C1, C2, D2, E1, F1, G1, and G3) developed CEP on C6/36 cells after three blind passages. The CPE-positive cells were subjected to detection of arboviruses by qRT-PCR. Of 12 arboviruses targeted, JEV was detectable in the cells inoculated with C1, C2, D2, E1, F1, and G1 samples (data not shown), in line with the results of JEV detection performed on BHK-21 cells (Figure 5 and Supplementary Table 6), whereas GETV was detected in the cells inoculated with B3, C1, and E1 samples (Supplementary Table 6). Interestingly, both JEV and GETV were detected in the cells inoculated with C1 and E1 samples, suggesting the copresence of these two viruses in mosquitoes. The supernatants of C6/36 cells inoculated with B3, C1, and E1 samples were further inoculated onto BHK-21 cells and subjected to detection of the presence of GETV by IFA and Western blot analysis. GETV was detectable in the inoculated cells, as demonstrated by IFA (Figure 6A) and Western blot (Figure 6B) with antibodies specific to E2 protein of GETV. To further confirm these results, the E2 gene of GETV was amplified from the inoculated BHK-21 cells and sequenced. The resulting sequences (GETV-B3/YN, GETV-C1/YN, and GETV-E1/YN) were deposited in GenBank and subsequently used to construct the Bayesian phylogenetic tree. The generated Bayesian phylogenetic tree indicated that the GETV detected in the B3, C1, and E1 samples were closely matched with the previously reported GETV strains (Figure 7).

GETV, a positive sense ssRNA virus belonging to the *Alphavirus* genus of the *Togaviridae* family, is a mosquito-borne virus that is originally isolated from *Cx.* mosquitoes in Malaysia in 1955. It has a broad geographical distribution and can infect humans, monkeys, cattle, birds, pigs, horses, and other mammals (Fukunaga et al., 2000; Bryant et al., 2005; Bannai et al., 2015). GETV is known to be pathogenic to horses and pigs and cause economic losses. In China, GETV is first identified in Hainan province in the southern China in 1964 from wild-caught mosquitoes, and since then, several studies have reported its presence in China with a wide distribution (Zhai et al., 2008; Feng et al., 2012). A recent study provides an evidence of the first GETV outbreak in piglets in Hunan province of China (Yang et al., 2018). In addition, GETV emergence in racehorse in an equestrian training center in Guangdong province in the southern China (Lu et al., 2019) and in cattle in the northeastern region of China has been reported (Liu et al., 2019).

In the present study, the GETV-positive samples were collected from Ruili and Longchuan farms, showing the presence of this virus in these areas. A recent serological survey of GETV in various domestic animals in Yunnan province showed that the positive rates of GETV neutralizing antibodies varied from 2 to 79% in domestic animals (chicken 2%, ducks 6%, pigs 46%, and beef cattle 79%) (Li et al., 2019). Notably, pigs and beef cattle have relatively higher antibody titers, which might be acting as a potential host for this virus. However, this speculation needs to be proven with further experiments. Furthermore, pigs and cattle are the most common breeding species in Yunnan’s vast rural areas, and GETV may have a large number of host animals in this region. In the present study, the GETV-positive mosquito samples were collected from swine and buffalo/cattle farms (Supplementary Table 2), which provided a potential evidence of GETV circulation in animals.

GETV has been isolated from *Ar. subalbatus* in Yunnan province (Zhai et al., 2008), and GETV-C1/YN and GETV-E1/YN strains detected in the present study were also located in the same evolutionary branch matching to Yunnan strains (Figure 7). However, GETV-B3/YN strain was clustering with a GETV strain (AY702913) that is reported from South Korea. These findings suggest that diverse GETV populations with different evolutionary states exist in Yunnan province. Therefore, strengthening the detection and monitoring of GETV infections is essential to prevent future epidemic in this region.

A number of GETV strains have been isolated across the latitude 19°N (Hainan province) to 42°N (Liaoning province) and longitudes 97°E (Gansu province) to 124°E (Liaoning province) from *Cx*., *Ae*., *Ar*., and *An*. mosquitoes (Zhai et al., 2008; Li et al., 2017). Together with these previous reports, the presence of GETV in mosquitoes from different animal farms indicates a threat to animal health, particularly horses, pigs, and cattle. Monitoring of GETV in animals is necessary along with taking precautionary measures, such as mosquito control, to prevent the related animal diseases in China and reduce economic losses.

### Family *Parvoviridae*, Parvoviruses

**FIGURE 8:**
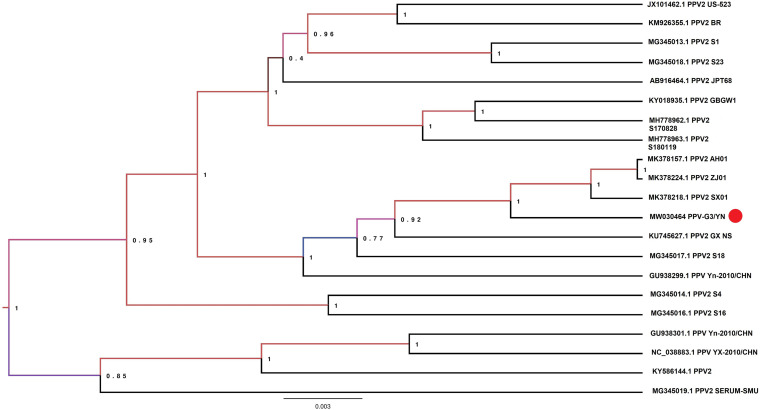
Phylogenetic analysis of nucleotide sequences of NS1 gene of PPV2 detected in the mosquito samples. The PPV 2 sequences identified in the present study are labeled with red circle.

**FIGURE 9:**
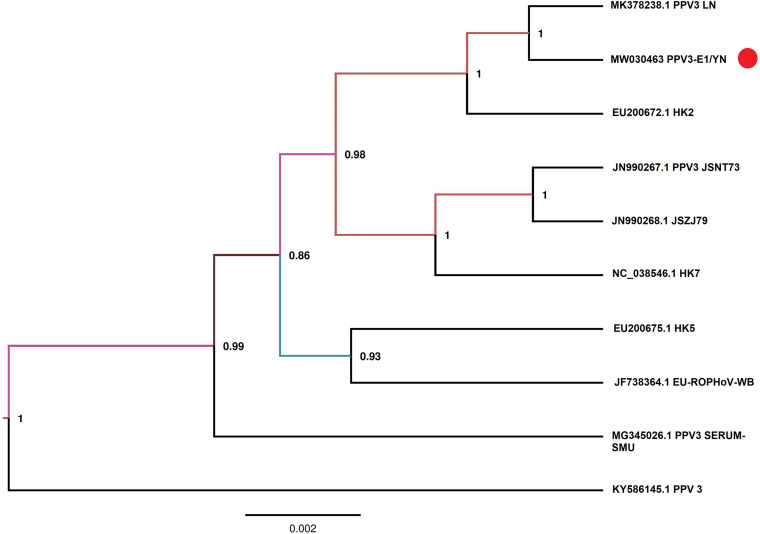
Phylogenetic analysis of nucleotide sequences of NS1 gene of PPV3 detected in the mosquito samples. The PPV sequences identified in the present study are labeled with red circle.

Samples E1 and G3 contained considerable reads closely related to the subfamily *Parvovirinae*, family *Parvoviridae* (Figure 2 and Table 3). Blast results revealed that these viral sequences matched more closely with PPV2 and PPV3. The PPV sequence detected in each mosquito sample was named accordingly (Table 3). The reads related to PPVs were assembled into contigs and then blasted against the reference PPV isolates. The contigs assembled from G3 and E1 samples shared 99.1 and 99.5% nucleotide similarity with the reference PPV2 and PPV3 strains, respectively (Table 3). Phylogenetic analysis based on the complete NS1 gene sequences indicated that all the detected PPV sequences had close relationship with the already known parvoviruses (Figures 8, 9).

**TABLE 3:** Information of assembled contigs of PPVs and HMV2.

Virus	Samples	Contigs	No. of reads	Matched strains (Genbank no.)	Query cover	Identity (%)	*E*-value	Covered range	Covered viral gene	Sequences for phylogenetic analysis	Name of strain
PPV2	G3	1	46	MK378224	100	99.12	0	2,078–4,802	NS1-Cap	NS1	PPV-G3/YN
		2	44	MK378224	100	99.12	0	148–1,545	NS1		
		3	70	MK378224	100	99.12	0	5,129–5,227	Cap		
PPV3	E1	1	1,004	MK378238	100	99.52	0	2,612–4,714	NS1-Cap	NS1	PPV-E1/YN
		2	542	MK378238	100	99.52	0	27–1,648	NS1		
		3	5	MK378238	100	99.52	0	1,882–2,111	NS1		
HMV2	B3	1	28,323	KX882764	100	95.96	0	86–2,784	Hypothetical protein 1 and RdRp in segment 1	Hypothetical protein 1	HMV-B3/YN
	C1	1	2,673,369	KX882764	100	94.64	0	1–3,058			HMV-C1/YN
	C2	1	269,335	KX882764	100	94.88	0	1–3,045			HMV-C2/YN
	C3	1	2,422	KX882873	100	94.64	0	1–2,770			HMV-C3/YN
	D2	1	28,451	KX882764	100	95.12	0	1–2,785			HMV-D2/YN
	E1	1	44,389	KX882764	100	94.64	0	16–2,924			HMV-E1/YN
	F1	1	4,353	KX882873	100	95.53	0	70–2,910			HMV-F1/YN
	G1	1	2,115,002	KX882832	99	96.54	0	13–3,055			HMV-G1/YN
	G2	1	658	KX882764	100	97.01	0	215–2,522			HMV-G2/YN
	G3	1	28,144	KX882873	100	96.26	0	8–2,810			HMV-G3/YN
HMV2	B3	1	26,113	KX882765	100	99.02	0	17–1,651	Hypothetical protein in segment 2		
	C1	1	2,120,880	KX882765	100	99.03	0	193–1,641			
	C2	1	132,560	KX882765	100	99.20	0	17–1,645			
	C3	1	10,258	KX882765	100	99.34	0	10–1,372			
	D2	1	78,017	KX882765	100	99.34	0	17–1,647			
	E1	1	40,696	KX882765	100	97.34	0	17–1,647			
	F1	1	4,547	KX882765	100	97.12	0	17–1,647			
	G1	1	1,562,970	KX882765	100	97.10	0	161–1,847			
	G2	1	623	KX882765	100	97.16	0	17–1,636			
	G3	1	18,620	KX882765	100	97.04	0	14–1,636			

PPVs are small, non-enveloped, and single-stranded DNA viruses and ubiquitous in the global pig population (Cui et al., 2017; Xing et al., 2018). These viruses are host-specific to pigs, and infection of PPVs can result in a persistent viremia (Cságola et al., 2016) and therefore provides mosquitoes a longer time to take PPVs during blood feeding. The role of mosquitoes as a biological or mechanical vector has not been defined yet. Among PPVs, PPV1 causes major reproductive failure in sows (Karniychuk and Nauwynck, 2013) and is an economically important viral pathogen for pig industry worldwide (Zhou and Yang, 2010), whereas other PPVs are usually coinfected with other pathogens and may play associated role in disease development (Saekhow et al., 2016; Ghosh et al., 2018). PPVs have spread widely in Chinese swine herds and are also frequently reported in single infections or coinfections with other porcine viruses, such as porcine circovirus 2, porcine reproductive and respiratory virus, and torque teno sus virus 1a (Sun et al., 2015; Qin et al., 2018). In our previous investigation of mosquito virome from pig farms of Shanghai, we detected highly abundant reads of PPV2, PPV3, PPV4, PPV5, and PPV6 (Hameed et al., 2020). However, PPV reads detected in the present study were relatively less. This difference was probably due to difference in animal species in Yunnan farms, e.g., more buffalo/cattle population in Yunnan farms. It is known that mosquito is not a biological host of PPVs; therefore, we speculated that mosquitoes might take PPVs from viremic animals. However, further investigation is required to explore the transmission potential of mosquito whether as a mechanical or biological vector. In addition, mosquito control in pig farms should be applied along with general disease control strategies in the affected areas to reduce losses as a consequence of severe clinical signs and co-infections.

### Luteo-Like Virus, Hubei Mosquito Virus 2

**FIGURE 10:**
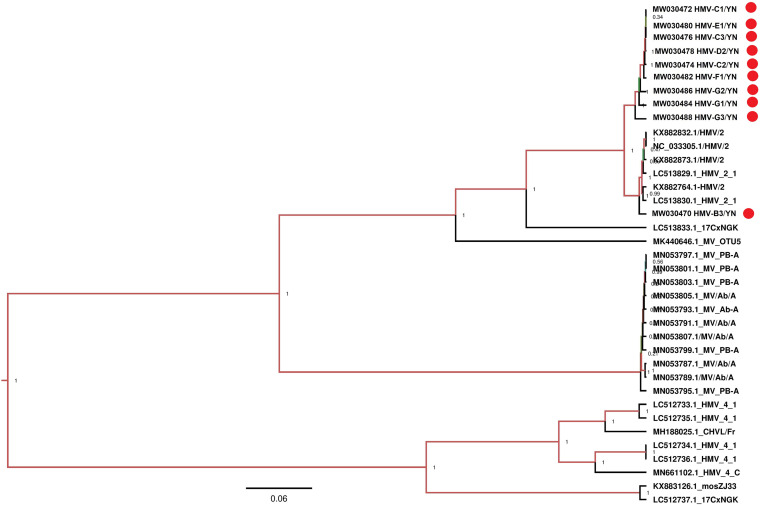
Phylogenetic analysis of nucleotide sequences of hypothetical protein 1 gene of HMV2 detected in mosquito samples. Different HMV-2 sequences identified in the present study are labeled with red circle.

Notably, all mosquito samples collected from different locations (Table 1) contained a large number of viral reads matched with HMV2 that is a luteo-like virus. Luteoviruses are commonly known to include many economically important plant pathogens (Ali et al., 2014). Recently, a number of luteo-like viruses have been discovered in diverse invertebrates, such as dragonflies, spiders, and also in mosquitoes, indicating that this group of viruses has wider range of hosts than previously known (Pettersson et al., 2017; Shi et al., 2017; Faizah et al., 2020). The reads related to HMV2 were aligned with segments 1 and 2 of HMV2 and assembled into contigs with variable lengths (Table 3). All these contigs showed 94.6–99.3% nucleotide identity to the respective segments of referenced HMV2 strains. The HMV2 sequence detected in each mosquito sample was named accordingly (Table 3). Phylogenetic analysis based on the hypothetical protein 1 gene sequence of HMV2 indicated that the observed HMV2 from all samples shared a common ancestor back to *Cx. inatomii* luteo-like virus (LC 513833.1 strain) (Figure 10), which is reported recently in *Cx.* mosquitoes from Japan (Faizah et al., 2020). In a recent study from Hubei province of China, HMV2 is present in the four most common mosquito species of China (Xia et al., 2018), which was in line with our findings.

Under field conditions, mosquitoes naturally infected with insect-specific viruses may be superinfected with arboviruses, such as JEV, DENV, WNV, etc., after feeding upon infected vertebrate hosts. This coinfection might increase or decrease the competence or fitness of arboviruses. Multiple studies have demonstrated that coinfection of insect-specific viruses may have an impact on arboviruses transmission both *in vitro* and *in vivo*, as well as on vector competence of arboviruses. Kuwata et al. (2015) reported that superinfection of JEV might be harmful to CxFV-infected *Cx. tritaeniorhyncus* mosquitoes by using CxFV-infected *Cx. tritaeniorhyncus* Giles cell line, although this study is unable to demonstrate the correlation between CxFV occurrence and JEV infection. On contrary to this, Fredericks et al. and other previous studies suggest that the persistent insect-specific virus infections encountered by arboviruses in nature and in mosquitoes hypothetically infected for environmental release may not reduce and may, in fact, enhance arboviruses transmission (Hall-Mendelin et al., 2016; Parry and Asgari, 2018; Fredericks et al., 2019). JEV has a reputable endemic history in Yunnan province, and the presence of HMV2 in mosquitoes might impact JEV transmission. Clearly, however, more extensive studies need to be undertaken for the elucidation of HMV2 infection in mosquitoes that might have an impact on arboviruses transmission.

### Non-classified Viruses

Currently, little information is available about the non-classified viruses and their evolution in mosquitoes. In the present study, a considerable number of reads were classified as non-classified virus sequences (Figure 2), presumably belonging to unexplored novel viruses, which have not been studied in the past. The discovery and characterization of these non-classified viruses can provide valuable information on their genetic diversity, ecology, evolution, and potential to threaten animal and human health. Additionally, as viruses have extraordinary mutation potential, which is leading to the emergence of new vertebrate diseases (Sanjuán et al., 2010; Greenwood et al., 2018; Kretova et al., 2018), so the discovery and surveillance of these viruses can also provide useful information to prevent future outbreaks.

## Conclusion

The virome burden in mosquitoes collected from different animal farms located at Yunnan province of China was examined by using mNGS. The generated viral metagenomic data revealed that the viral community in mosquitoes was highly diverse and varied in abundance among animal farms, which contained more than 19 viral taxonomic families. Notably, the presence of viral reads matched with JEV, GETV, and PPVs in mosquitoes collected from different geographic sites suggested a potential circulation of these viruses in their vertebrate hosts. Overall, based on the present information, special attention to mosquito control in animal farms and new approaches to prevent microbial threats from mosquitoes should be merited.

## Data Availability Statement

The datasets presented in this study can be found in online repositories. The names of the repository/repositories and accession number(s) can be found below: MW030461 to MW030494.

## Author Contributions

All authors have directly participated in the planning, execution, or analysis of the study and resulting manuscript, and have read and approved the version of the article submitted. And our institute has also given the agreement to submission. No part of this manuscript has been published or submitted elsewhere.

## Conflict of Interest

The authors declare that the research was conducted in the absence of any commercial or financial relationships that could be construed as a potential conflict of interest.

## Footnotes

1
https://www.ncbi.nlm.nih.gov/genbank/

2
http://tree.bio.ed.ac.uk/software/figtree/

## Supplementary Material

Supplementary Figure 1 Maximum likelihood phylogenetic tree based on COI gene sequences of different mosquitoes collected from animal farms. Sequence analysis was conducted using MEGA version 7.0 software with 1,000 replications. The identified mosquito species are boxed with different colors.

Supplementary Figure 2 Relative abundance of viral reads from mosquito samples.

Supplementary Table 1 Information of animal farms for mosquito collection.

Supplementary Table 2 Profile of mosquitoes collected in this study.

Supplementary Table 3 Primer sequence used for detection of arboviruses.

Supplementary Table 4 Percentage of reads related to specific viruses.

Supplementary Table 5 JEV isolates analyzed in this study.

Supplementary Table 6 Ct value of qRT-PCR for detection of arboviruses.
