# Corrigendum: A metagenomic analysis of mosquito virome collected from different animal farms at Yunnan–Myanmar border of China

**DOI:** 10.3389/fmicb.2024.1393495

**Published:** 2024-03-19

**Authors:** Muddassar Hameed, Abdul Wahaab, Tongling Shan, Xin Wang, Sawar Khan, Di Di, Liu Xiqian, Jun-Jie Zhang, Muhammad Naveed Anwar, Mohsin Nawaz, Beibei Li, Ke Liu, Donghua Shao, Yafeng Qiu, Jianchao Wei, Zhiyong Ma

**Affiliations:** Shanghai Veterinary Research Institute, Chinese Academy of Agricultural Sciences, Shanghai, China

**Keywords:** mosquito, mosquito virome, metagenomics, viral community, animal farm

In the published article, there was an error in Figure 5 as published. When preparing Figure 5A, since JEV-C1 and JEV-G1 are on two PPT pages, when copying JEV-G1, in order to keep the zoom ratio of the two pages, JEV-C1 is directly copied to the second page as a contrast, so there is confusion when inserting the JEV-G1 DAPI picture. The corrected Figure 5 and its caption appear below.

**Figure 5:**
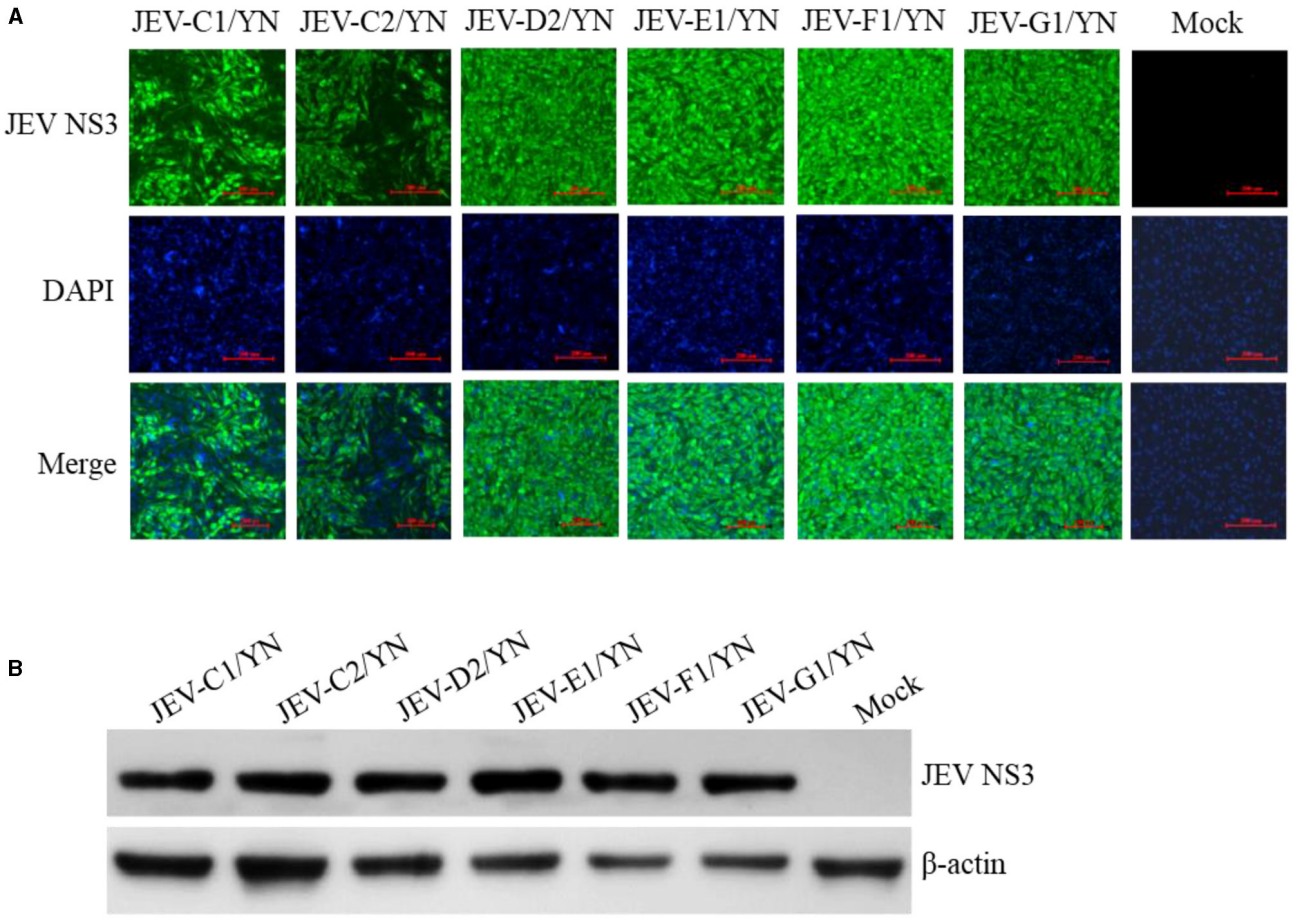
Detection of JEV in BHK-21 cells. BHK-21 cells were inoculated with the supernatants harvested from the CPE-positive BHK-21 cells inoculated with mosquito samples and incubated for 36 h. The presence of JEV was detected by IFA **(A)** and Western blot **(B)** with antibodies specific to JEV NS3 protein. Nuclei were stained with DAPI.

The authors apologize for this error and state that this does not change the scientific conclusions of the article in any way. The original article has been updated.

