# Supplementary figures and images for: A Metagenomic Analysis of Mosquito Virome Collected From Different Animal Farms at Yunnan–Myanmar Border of China
Source: Front Microbiol. 2021 Feb 8;11:591478. doi: 10.3389/fmicb.2020.591478 (PMC7898981; doi:10.3389/fmicb.2020.591478)

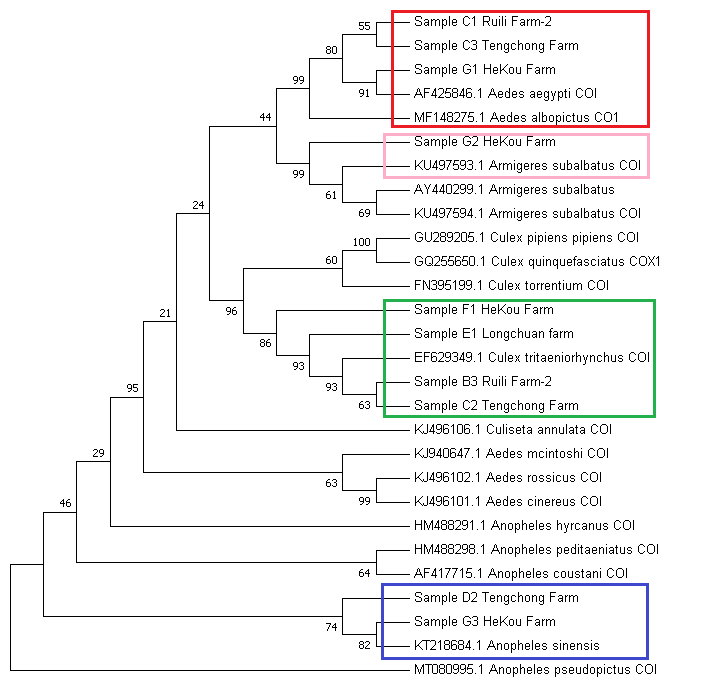

Supplement: Supplementary Figure 1 — Maximum likelihood phylogenetic tree based on COI gene sequences of different mosquitoes collected from animal farms. Sequence analysis was conducted using MEGA version 7.0 software with 1,000 replications. The identified mosquito species are boxed with different colors. [file Image_1.PNG]

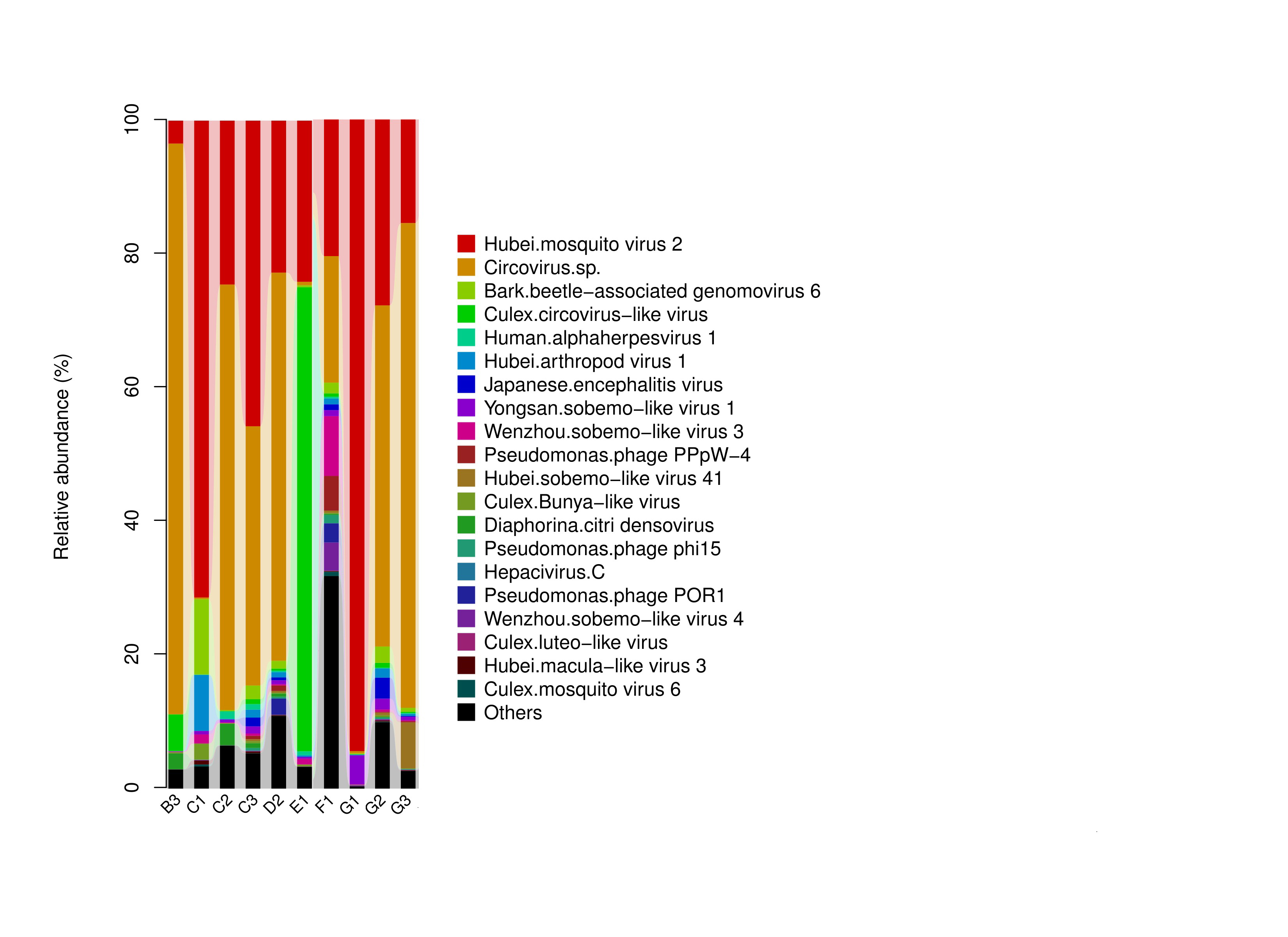

Supplement: Supplementary Figure 2 — Relative abundance of viral reads from mosquito samples. [file Image_2.JPEG]
