# Supplementary material for: A Metagenomic Analysis of Mosquito Virome Collected From Different Animal Farms at Yunnan–Myanmar Border of China
Source: Front Microbiol. 2021 Feb 8;11:591478. doi: 10.3389/fmicb.2020.591478 (PMC7898981; doi:10.3389/fmicb.2020.591478)
Supplement: Supplementary Table 1 — Information of animal farms for mosquito collection. [file Table_1.DOCX]

**Supplementary Table 1**. Information of animal farms for mosquito collection

| County | Farm | Average temperature | Altitude（m） | Longitude | Latitude | Animal | Farm size | Health status |
| --- | --- | --- | --- | --- | --- | --- | --- | --- |
| Tengchong (TC) | BFL | 19℃-24℃ | 1677 | 98.382294 | 25.024549 | Cattle/Buffalo | >500 | good |
| Longchuan (LC) | YEY | 22℃-29℃ | 965 | 97.962967 | 24.35891 | Swine | >3000 | good |
| Ruili (RL) | YPY | 23℃-30℃ | 778 | 97.511913 | 24.011498 | Cattle/Buffalo | >5000 | good |
| Hekou (HK) | MYJ | 25℃-32℃ | 110 | 103.967567 | 22.52866 | Swine | >2000 | good |
