# Supplementary material for: A Metagenomic Analysis of Mosquito Virome Collected From Different Animal Farms at Yunnan–Myanmar Border of China
Source: Front Microbiol. 2021 Feb 8;11:591478. doi: 10.3389/fmicb.2020.591478 (PMC7898981; doi:10.3389/fmicb.2020.591478)
Supplement: Supplementary Table 2 — Profile of mosquitoes collected in this study. [file Table_2.DOCX]

**Supplementary Table 2.** Profile of mosquitoes collected in this study

| County | Farm | Animal | *Cx. tritaeniorhyncus* | *Cx. quinquefasciatu* | *An. sinensis* | *An. Minimus* | *Ar. subalbatus* | *Ar. obturbans* | *Ae. aegypti* | Total |
| --- | --- | --- | --- | --- | --- | --- | --- | --- | --- | --- |
| Tengchong (TC) | BFL | Cattle/Buffalo | 1269 (♂200, ♀1069) | 0 (♂0, ♀0) | 115 (♂16, ♀99) | 0 (♂0, ♀0) | 0 (♂0, ♀0) | 0 (♂0, ♀0) | 690 (♂196, ♀504) | 2074 |
| Longchuan (LC) | YEY | Swine | 340 (♂80, ♀260) | 5 (♂0, ♀5) | 0 (♂0, ♀0) | 2 (♂0, ♀2) | 0 (♂0, ♀0) | 0(♂0, ♀0) | 250 (♂50, ♀200) | 597 |
| Ruili (RL) | YPY | Cattle/Buffalo | 767 (♂163, ♀604) | 0 (♂0, ♀0) | 110 (♂16, ♀94) | 4 (♂1, ♀3) | 0 (♂0, ♀0) | 6 (♂0, ♀6) | 450 (♂86, ♀364) | 1337 |
| Hekou (HK) | MYJ | Swine | 231 (♂43, ♀188) | 3 (♂0, ♀3) | 118 (♂11, ♀107) | 0(♂0, ♀0) | 101 (♂5, ♀96) | 4 (♂0, ♀4) | 111 (♂4, ♀107) | 568 |
| Total | | | 2607 | 8 | 343 | 6 | 101 | 10 | 1501 | 4576 |
| % | | | 56.97% | 0.17% | 7.50% | 0.13% | 2.21% | 0.22% | 32.80% |  |
