# Supplementary material for: A Metagenomic Analysis of Mosquito Virome Collected From Different Animal Farms at Yunnan–Myanmar Border of China
Source: Front Microbiol. 2021 Feb 8;11:591478. doi: 10.3389/fmicb.2020.591478 (PMC7898981; doi:10.3389/fmicb.2020.591478)
Supplement: Supplementary Table 3 — Primer sequence used for detection of arboviruses. [file Table_3.DOCX]

**Supplementary Table 3.** Primer sequence used for detection of arboviruses

| Primer name | Sequence (5́-3́) | Product size (bp) | Targeted gene |
| --- | --- | --- | --- |
| JEV-C1 | TTCACGAACATTGCCGTCCA | 781 | NS5 |
|  | ACTTGGTTTATTGCCGCGT |  |  |
| JEV-C2 | ATTGGACCACAACACTTGGA | 778 | NS5 |
|  | AGTGAGGTCATGTAGTCA |  |  |
| JEV-D2 | ACACATTCACGAACATTGCT | 1004 | NS5 |
|  | GTAGTCAACATAATTTTC |  |  |
| JEV-E1 | ATTGGACCACAACACTTG | 922 | NS5 |
|  | GTATCCGGTGGCAGCTTT |  |  |
| JEV-F1 | TTCACGAACATTGCCGTCCA | 781 | NS5 |
|  | ACTTGGTTTATTGCCGCGT |  |  |
| JEV-G1 | ATTGGACCACAACACTTGGA | 778 | NS5 |
|  | AGTGAGGTCATGTAGTCA |  |  |
| GETV E2 | AAGTGCCATGCACAACGTAC | 403 | E2 |
|  | ACTGTCAGTTCTCTCTTTCCGT |  |  |
| Bunyavirus | ATGACTGAGTTGGAGTTTGATGTCGC | 251 | C |
|  | TGT TCC TGT TGC CAG GAA AAT |  |  |
| Banna virus | GTGACGATAACGCCAAGT | 260 | seg 10 |
|  | TTTCCAGTCCCAAGAGCC |  |  |
| *Culex* flavivirus | AACGGACTTCTTGAGTTTCGC | 1200 | E |
|  | GCCTTGGTGTAGACAAAGTATC |  |  |
| Chickengunya virus | CGTGGTGTACAAAGGTGACG | 646 | E1 |
|  | ACGCCGGGTAGTTGACTATG |  |  |
| Kadipiro virus | GAC GCT TTG AGA TTA TCT CGA C | 354 | VP12 |
|  | GCT CAA TCG CAT TCT CAC C |  |  |
| Liaoning virus | GGAAGAATCAATGCCGTAGCCAC | 505 | VP12 |
|  | GTGACGATCTTCTCTGAACCAGTG |  |  |
| Yunnan orbivirus | AGCATTCGGTACGCAGTATCTCG | 831 | NS2 |
|  | GCCGAGCCGATCATGTCACGTGT |  |  |
| Dengue virus | GTGACAATGCTCCTTATGCTGCTG | 351 | C/PrM |
|  | TCCAGGCACCTTCAGAGGACATC |  |  |
| Zika virus | CCTTGGATTCTTGAACGAGGA | 192 | NS5 |
|  | AGAGCTTCATTCTCCAGATCAA |  |  |
| West Nile virus | AAAGGCATTGGTAAGATTG | 443 | NS5 |
|  | ATTGAAGTCAGCGAAGTGT |  |  |
| JEV (qRT-PCR) | TACAACATGATGGGAAAAAGAGAGAAGAA | 225 | NS5 |
|  | CTTGCTTTCCTGCTATGTCACGGAGGA |  |  |
| GETV (qRT-PCR) | GAGAAAGAAGTGCTTGC | 189 | nsP1 |
|  | GGT GATCTTCTTTACCA |  |  |
