# Supplementary material for: A Metagenomic Analysis of Mosquito Virome Collected From Different Animal Farms at Yunnan–Myanmar Border of China
Source: Front Microbiol. 2021 Feb 8;11:591478. doi: 10.3389/fmicb.2020.591478 (PMC7898981; doi:10.3389/fmicb.2020.591478)
Supplement: Supplementary Table 5 — JEV isolates analyzed in this study. [file Table_5.DOCX]

**Supplementary Table 5.** JEV isolates analyzed in this study

| Strain | Date | Country/Location | Host | Genotype | GenBank accession no. |
| --- | --- | --- | --- | --- | --- |
| JaTAn1/90 | 1990 | Japan | pig | III | AB551991 |
| B-0860/82 | 1982 | Thailand | pig | I | GQ902058 |
| B-1381-85 | 1985 | Thailand | pig | I | GQ902061 |
| 90VN70 | 1990 | Vietnam | Human | I | HM228921 |
| Ishikawa | 1994 | Japan | Mosquitoes | I | AB051292 |
| YN82BN8219 | 1982 | China-Yunan | Mosquitoes | I | JN381834 |
| K94P05 | 1994 | Korea | Mosquitoes |  | AF045551 |
| M28 | 1977 | China | Mosquitoes | I | KT957422 |
| BN82215 | 1982 | China | Mosquitoes | I | KT957423 |
| KV1899 | 1999 | Korea | Pig | I | AY316157 |
| YN79Bao83 | 1979 | China-Yunan | Mosquitoes | I | JN381851 |
| Sw-Tokyo-373-2005 | 2005 | Japan | Pig | I | AB698907 |
| Sw-Mie-34-2004 | 2004 | Japan | Pig | I | AB698909 |
| 131V | 2007 | China | Human | I | GU205163 |
| JX61 | 2008 | China | Pig | I | GU556217 |
| HEN0701 | 2007 | China | Pig | I | FJ495189 |
| GZ56 | 2008 | China-GuiZhou | Human | I | HM366552 |
| HN0411 | 2004 | China-Henan | Mosquitoes | I | JN381831.1 |
| JEV/sw/Mie/40/2004 | 2004 | Japan | Pig | I | AB241118.1 |
| JEV/sw/Mie/41/2002 | 2002 | Japan | Pig | I | AB241119.1 |
| TC2009-3 | 2009 | Taiwan | Mosquitoes | I | JF499788.1 |
| YN0967 | 2009 | China-Yunan | Mosquitoes | I | JF706268.1 |
| BL06-50 | 2006 | China-Guangxi | Mosquitoes | I | JF706270.1 |
| LN02-102 | 2002 | China-Lioning | Mosquitoes | I | JF706278.1 |
| YN05124 | 2005 | China-Yunan | Mosquitoes | I | JF706281.1 |
| SD0810 | 2008 | China-Shandong | Mosquitoes | I | JF706286.1 |
| YN0623 | 2006 | China-Yunan | Mosquitoes | I | JN381836.1 |
| SH03103 | 2003 | China-Shanghai | Mosquitoes | I | JN381847.1 |
| SH53 | 2001 | China-Shanghai | Mosquitoes | I | JN381850.1 |
| JEV/Taiwan/TPC0906ah/M/2009 | 2009 | Taiwan | Mosquitoes | I | KF667318.1 |
| JEV/Taiwan/TC1006h/M/2010 | 2010 | Taiwan | Mosquitoes | I | KF667321.1 |
| DH10M978 | 2010 | China | Mosquitoes | I | KT229573.1 |
| YN09M57 | 2009 | China | Mosquitoes | I | KT229574.1 |
| YNTC07172 | 2007 | China | Mosquitoes | I | KT957419.1 |
| LN02-102 | 2002 | China-Lioning | Mosquitoes | I | JF706278.1 |
| SC0415 | 2004 | China-Sichuan | Mosquitoes | I | JN381838.1 |
| SH17M-07 | 2007 | China |  | I | EU429297.1 |
| SH80 | 2001 | China-Shanghai | Mosquitoes | I | JN381848.1 |
| SX09S-01 | 2009 | China | Pig | I | HQ893545.1 |
| XJ69 | 2007 | China | Mosquitoes | I | EU880214.1 |
| XZ0938 | 2009 | China-Xizhang | Mosquitoes | I | HQ652538.1 |
| YN05155 | 2005 | China-Yunan | Mosquitoes | I | JN381852.1 |
| JEV/sw/Okinawa/127/2012 | 2012 | Japan | Pig | I | AB920399 |
| JEV/MQ/Yamaguchi/2013-2 | 2013 | Japan | Mosquitoes | I | AB981184.1 |
| JEV/Taiwan/YL1206a/M/2012(2) | 2012 | Taiwan | Mosquitoes | I | KF667323.1 |
| JEV/Taiwan/YL1106b/M/2011(2) | 2011 | Taiwan | Mosquitoes | I | KF667327.1 |
| SCMY | 2014 | China | Pig | I | KU351668.1 |
| JS-1 | 2015 | China | Mosquitoes | I | KX357114.1 |
| SH7 | 2016 | China-Shanghai | Mosquitoes | I | MH753129.1 |
| SH2 | 2016 | China-Shanghai | Mosquitoes | I | MH753133.1 |
| FU | 1995 | Australia | Human | II | AF217620 |
| JKT6468 | 1981 | Indonesia | Mosquitoes | IV | AY184212 |
| Tengah | 1952 | Singapore | Human | V | KM677246 |
| Muar | 1952 | Malaysia | Human | V | HM596272 |
| WTP-70-22 | 1970 | Malaysia | Mosquitoes | II | HQ223286 |
| XZ0934 | 2009 | China-Tibet | Mosquitoes | V | JF915894 |
| JaGAr 01 | 1959 | Japan | Mosquitoes | III | AF069076 |
| HVI | 1959 | Taiwan | Mosquitoes | III | AF098735 |
| CH13 | 1957 | China-Sichuan | Human | III | JN381870 |
| Nakayama | 1935 | Japan | Human | III | EF571853 |
| YLG | 1955 | China-Fujian | Human | III | JF706280 |
| ZMT | 1955 | China-Fujian | Human | III | JF706283 |
| ZSZ | 1955 | China-Fujian | Human | III | JN381862 |
| CZX | 1954 | China-Fujian | Human | III | JN381865 |
| LYZ | 1957 | China-Fujian | Human | III | JN381869 |
| YN | 1954 | China-Yunan | Human | III | JN381871 |
| p3 | 1949 | China | Human | III | JEU47032 |
| Vellore P20778 | 1958 | India | Human | III | AF080251 |
| JaTH160 | 1960 | Japan | Human | III | AB269326.1 |
| JaTAn1/75 | 1975 | Japan | Pig | III | AB551990 |
| TL | 1965 | Taiwan | Mosquitoes | III | AF098737 |
| JaOH0566/Japan/1966/human | 1966 | Japan | Human | III | AY508813 |
| GP78 | 1978 | India | Human | III | AF075723 |
| GSS | 1960 | China-Beijing | Human | III | JF706275 |
| HYZ | 1979 | China-Yunan | Human | III | JN381853 |
| TLA | 1971 | China-Lioning | Human | III | JN381868 |
| Ha3 | 1960 | China-Heilongjiang | Human | III | JN381872 |
| Ling | 1965 | Taiwan | Human | III | L78128 |
| RP 2ms | 1985 | Taiwan | Mosquitoes | III | AF014160 |
| RP9 | 1985 | Taiwan | Mosquitoes | III | AF014161 |
| K87P39 | 1987 | South Korea | Mosquitoes | III | AY585242 |
| CH1392 | 1990 | Taiwan | Mosquitoes | III | AF254452 |
| KPP82-39-214CT | 1982 | Thailand | Mosquitoes | III | GQ902063 |
| JaOArS982 | 1982 | Japan | Mosquitoes | III | M18370 |
| SH3 | 1987 | China-Shanghai | Human | III | JN381864 |
| DH107 | 1989 | China-Yunan | Mosquitoes | III | JN381873 |
| K88A071 | 1988 | South Korea | Mosquitoes | III | KR908703 |
| JaTAn2/91 | 1991 | Japan | Pig | III | AB551992 |
| T1P1 | 1997 | Taiwan | Mosquitoes | III | AF254453 |
| 04940-4 | 2002 | India | Mosquitoes | III | EF623989 |
| 014178 | 2001 | India | Human | III | EF623987 |
| 057434 | 2005 | India | Human | III | EF623988 |
| DL0445 | 2004 | China-Yunan | Mosquitoes | III | JN381854 |
| HLJ02-134 | 2002 | China- Heilongjiang | Culicoides | III | JF706276 |
| DL04-29 | 2004 | China-Yunan | Mosquitoes | III | JF706272 |
| Fj02-29 | 2002 | China-Fujian | Human | III | JF706273 |
| JH0418 | 2004 | China-Yunan | Mosquitoes | III | JN381855 |
| GZ042 | 2004 | China-GuiZhou | Mosquitoes | III | JN381857 |
| YN98A151 | 2003 | China-Yunan | Mosquitoes | III | JN381861 |
| SH045 | 2004 | China-Shanghai | Mosquitoes | III | JN381866 |
| Fj0276 | 2002 | China-Fujian | Human | III | JN381867 |
| JEV/SW/GZ/09/2004 | 2004 | China | Pig | III | KF297916 |
| JEV/Taiwan/TP0506a/M/2005 | 2005 | Taiwan | Mosquitoes | III | KF667310 |
| YUNNAN0901 | 2009 | China | Mosquitoes | III | JQ086762 |
| YUNNAN0902 | 2009 | China | Pig | III | JQ086763 |
| IND-WB-JE1 | 2008 | India | Human | III | JX050179 |
| IND-WB-JE2 | 2010 | India | Human | III | JX072965 |
| JEV/eq/India/H225/2009 | 2009 | India | Horse | III | JX131374 |
| CQ11-66 | 2010 | China | Human | III | KC183732 |
| GZ | 2010 | China | Pig | III | KC915016 |
| JEV/SW/GD/01/2009 | 2009 | China | Pig | III | KF297915 |
| JEV/Taiwan/CSF-C2522/H/2006 | 2006 | Taiwan | Human | III | KF667311 |
| JEV/Taiwan/TPC0706a/M/2007 | 2007 | Taiwan | Mosquitoes | III | KF667312 |
| JEV/Taiwan/TC1006a/M/2010 | 2010 | Taiwan | Mosquitoes | III | KF667314 |
| JEV/Taiwan/YL0906c/M/2009(2) | 2009 | Taiwan | Mosquitoes | III | KF667315 |
| JEV/sw/GD/2008 | 2008 | China | Pig | III | KX965684 |
| SH0601 | 2006 | China | Pig | III | EF543861 |
| WHe | 2006 | China | Pig | III | EF107523 |
| JEV/SW/IVRI/395A/2014 | 2014 | India | Pig | III | KP164498 |
| SC201301 | 2013 | China | Pig | III | KU363309 |
| JEV/SC/2016-2 | 2016 | China | Pig | III | KX779520 |
| JEV/SC/2016-1 | 2016 | China | Pig | III | KX779521 |
| JEV/SC/2016-3 | 2016 | China | Pig | III | KX779522 |
| N28 | 2015 | China-Shanghai | Pig | III | MH753126 |
| SH1 | 2015 | China-Shanghai | Pig | III | MH753128 |
| SH15 | 2016 | China-Shanghai | Mosquitoes | III | MH753130 |
| SH19 | 2016 | China-Shanghai | Mosquitoes | III | MH753131 |
| SD12 | 2015 | China-Shanghai | Pig | I | MH753127 |
| SA14 | 1954 | China | Mosquitoes | III | U14163 |
