# Supplementary material for: A Metagenomic Analysis of Mosquito Virome Collected From Different Animal Farms at Yunnan–Myanmar Border of China
Source: Front Microbiol. 2021 Feb 8;11:591478. doi: 10.3389/fmicb.2020.591478 (PMC7898981; doi:10.3389/fmicb.2020.591478)
Supplement: Supplementary Table 6 — Ct value of qRT-PCR for detection of arboviruses. [file Table_6.DOCX]

**Supplementary Table 6.** Ct value of qRT-PCR for detection of arboviruses

| Target virus | Targeted gene | Sample for qRT-PCR | Mosquito sample | Name of strain | Ct value |
| --- | --- | --- | --- | --- | --- |
| JEV | NS5 | Samples collected from CPE-positive BHK-21 cells | C1 | JEV-C1/YN | 21.34 |
|  |  |  | C2 | JEV-C2/YN | 23.63 |
|  |  |  | D2 | JEV-D2/YN | 17.95 |
|  |  |  | E1 | JEV-E1/YN | 15.12 |
|  |  |  | F1 | JEV-F1/YN | 12.39 |
|  |  |  | G1 | JEV-G1/YN | 14.82 |
| GETV | nsP1 | Samples collected from CPE-positive C6/36 cells | B3 | GETV-B3/YN | 19.15 |
|  |  |  | C1 | GETV-C1/YN | 15.37 |
|  |  |  | E1 | GETV-E1/YN | 20.82 |
